# Quantification of cell-bubble interactions in a 3D engineered tissue phantom

**DOI:** 10.1038/s41598-017-06678-y

**Published:** 2017-07-24

**Authors:** C. Walsh, N. Ovenden, E. Stride, U. Cheema

**Affiliations:** 1 Centre for Mathematics and Physics in the Life Sciences and Experimental Biology (CoMPLEX), UCL Physics Building Gower Street, London, WC1E 6BT UK; 2 UCL Institute of Orthopaedics and Musculoskeletal Science, London, UK; 3 Department of Mathematics, University College London, London, UK; 4 Institute of Biomedical Engineering, Old Road Campus Research Building, University of Oxford, Oxford, UK

## Abstract

Understanding cell-bubble interactions is crucial for preventing bubble related pathologies and harnessing their potential therapeutic benefits. Bubbles can occur in the body as a result of therapeutic intravenous administration, surgery, infections or decompression. Subsequent interactions with living cells, may result in pathological responses such as decompression sickness (DCS). This work investigates the interactions that occur between bubbles formed during decompression and cells in a 3D engineered tissue phantom. Increasing the tissue phantoms’ cellular density resulted in decreased dissolved O_2_ (DO) concentrations (p = 0.0003) measured using real-time O_2_ monitoring. Direct microscopic observation of these phantoms, revealed a significant (p = 0.0024) corresponding reduction in bubble nucleation. No significant difference in growth rate or maximum size of the bubbles was measured (p = 0.99 and 0.23). These results show that bubble nucleation is dominated by DO concentration (affected by cellular metabolism), rather than potential nucleation sites provided by cell-surfaces. Consequent bubble growth depends not only on DO concentration but also on competition for dissolved gas. Cell death was found to significantly increase (p = 0.0116) following a bubble-forming decompression. By comparison to 2D experiments; the more biomimetic 3D geometry and extracellular matrix in this work, provide data more applicable for understanding and developing models of *in vivo* bubble dynamics.

## Introduction

The concentration of dissolved gas in any liquid is dependent upon the ambient pressure. A reduction in ambient pressure (decompression) reduces the amount of gas that can be held in solution and forces excess gas to form bubbles within the liquid. The process is ubiquitous occurring in volcanic eruptions^1^, carbonated beverages^2^, polymer foaming^3, 4^, water treatment^5^ and also in the human body. A rapid decompression may be experienced during ascent from a self contained underwater breathing apparatus (SCUBA) dive, pressurized construction works and space walks^6^. In these cases decompression leads to the formation of bubbles both in the blood stream and surrounding tissue^6, 7^. These bubbles interact with cells as well as larger biological structures, potentially causing a pathological response, known as Decompression sickness (DCS). Despite over 200 years of research the detailed mechanisms by which bubbles cause DCS are still unclear. The potential for bubbles to interact with almost any system in the body means DCS is a highly complex condition. This complexity coupled with the difficulties in measuring bubble dynamics *in vivo* has been a major research hurdle. In underwater diving various algorithms are used to prescribe safe ascent rate based on modelling the tissue gas dynamics of the particular dive^8^. However, every year recreational, military and commercial divers suffer from DCS, despite having ascended using such algorithms^9^. The long term health implications for such divers is unclear as is the effect of repetitive asymptomatic bubble formation that has been observed in many divers^10–12^. Consequently there is a need to improve our understanding of bubble-cell interactions with a view to better prevention and treatment of DCS. In addition, developing our understanding of bubble-cell interactions in the pathological case, will aid research into the potential therapeutic uses of bubbles^13^.

To address these research questions a system that enables quantification of bubble dynamics within a controllable biologically relevant model is needed. The need for control should be stressed as it enables hypotheses regarding biological and physical mechanisms of bubble insult to be tested in ways not possible in animal models or human divers due to practical and/or ethical considerations^14–16^.

The most widely used technique for detection of decompression induced bubbles *in vivo* is ultrasound. This technique enables the number of mobile vascular bubbles that pass the probe to be estimated by a grading system (venous gas embolism grade)^17^. It is however limited to measuring the vascular fraction of the bubble population^18^, and it is well documented that the venous gas embolism grade is a poor predictor of DCS symptom onset^6, 17, 19^. This poor predictive power may be due to the importance of the extravascular fraction of the bubble population, the importance of which has been demonstrated a small number of *in vivo* animal studies^20–23^. As extravascular bubbles cannot be easily measured *in vivo* and their involvement in DCS is widely accepted, dive algorithms predominantly model the dynamics of extravascular gas, either in solution or as bubbles^24^.

We have therefore developed a combination of an *in vitro* tissue phantom with a microscope compatible hyperbaric chamber that allows real-time quantification of bubble dynamics and measurement of cellular responses within a controlled and well-characterized tissue model.

Four interactions between cells, dissolved gas and bubbles were chosen for investigation with this system.

i) Cellular metabolism of oxygen, which reduces the total concentration of dissolved gas available for bubble formation and growth^6, 25^. This relationship was quantified through variation of dissolved oxygen concentrations (through increasing cell density) and measurement of subsequent bubble dynamics.
ii) Cell surfaces provide geometric stabilization for bubble micronuclei and hence are sites of bubble nucleation^26^. Quantification of bubble nucleation at varying cell densities was measured.
iii) Increased partial pressure of oxygen experienced at depth, causes cellular dysfunction and death^27, 28^. The viability of cells following decompression was measured after exposure to a known partial pressure of oxygen.
iv) Biomechanical and biochemical interactions of bubbles and cells results in cell death^29–33^. Cell viability following a bubble-forming decompression was measured.

Cellular metabolism of oxygen leads to an inherent pressure difference between arterial blood and tissues, this is a widely utilized effect in avoiding and treating DCS, known as the oxygen window. Any gas dissolves into a liquid according to Henry’s Law.

1 $${C}^{g}={k}_{h}^{g}p{p}^{g}$$

where *g* is the type of gas, *C* ^*g*^ is the concentration of the dissolved gas, ${k}_{h}^{g}$ is Henry’s solubility constant, and is specific to a given gas in a given liquid at a fixed temperature. *pp* ^*g*^ is the partial pressure of the gas (mole fraction of a gas/ total pressure). The partial pressure of any gas is the driving force for diffusion of that dissolved gas and the combined partial pressure of all dissolved gases, referred to as the tissue tension, is the driving force for bubble nucleation^34^.

The oxygen window occurs as CO_2_ is 20 times more soluble than O_2_ ${k}_{h}^{C{O}_{2}}=20{k}_{h}^{{O}_{2}}$. Thus, as O_2_ is metabolized to CO_2_, there is a deficit between the increase in ppCO_2_ and the decrease in ppO_2_. This results in a decrease in the tissue tension, reducing the probability of bubble nucleation^35^. In addition, as the amount of dissolved nitrogen does not change but the tissue tension decreases, the effect increases the *pp*N_2_ thereby increasing the gradient for nitrogen to diffuse out of any bubbles present^36^. Although all tissues in the body metabolise oxygen at different rates, the custom in dive algorithms is to have a single fixed value, implemented by grouping O_2_ and CO_2_ into a single fixed pressure contribution–the fixed metabolic gases^37, 38^. In other fields of research oxygen metabolism is more explicitly modelled by, Michaelis-Menten kinetics.

2 $$E+S\,\mathop{\mathop{\longleftrightarrow }\limits_{{k}_{s}}}\limits^{{k}_{f}}\,ES\,\mathop{\longrightarrow }\limits^{{k}_{cat}}\,P+E$$

where *E* is the enzyme, *S* is the substrate, *P* the product and *k* denotes a rate constant. *K*_*m*_, the Michaelis rate constant, can be written as *K*_*m*_ = (*k*_*r*_ + *k*_*cat*_)/*k*_*f*_. From this the rate of oxygen consumption (RO_2_) can be derived as:

3 $$R{O}_{2}=\frac{{V}_{max}p{p}^{{O}_{2}}}{p{p}^{{O}_{2}}+{K}_{m}}\frac{\sigma }{{k}_{h}^{{O}_{2}}}$$

*K*_*m*_ is often described as the half-maximal oxygen concentration and *V*_*max*_ is the maximum rate of oxygen consumption and *σ* is the cell density (cells/ml).

To model the spatial distribution of oxygen within a cellular tissue or tissue phantom, the Michaelis-Menten approximation can be incorporated into the diffusion equation.

4 $$\frac{\partial p{p}^{{O}_{2}}}{\partial t}=D{\nabla }^{2}\frac{\partial p{p}^{{O}_{2}}}{\partial x}-\frac{{V}_{max}p{p}^{{O}_{2}}}{p{p}^{{O}_{2}}+{K}_{m}}\frac{\sigma }{{k}_{h}^{{O}_{2}}}$$

where *D* is the diffusion coefficient of oxygen through the liquid and *x* is the spatial coordinate.

In this work the dissolved gas concentration is modelled using a diffusion equation with Michaelis-Menten kinetics for O_2_ and additional diffusion equation for N_2_ diffusion. (see methodology for full details). The simulated results were used to investigate the spatially explicit dissolved gas concentration in tissue phantoms of differing cell densities. Although oxygen is essential for respiration, high concentrations of oxygen may become toxic. At a cellular level, increased ppO_2_ increases the production of reactive oxygen species (ROS) such as the H_2_O_2_ and ${O}_{2}^{-}$. Increased levels of ROS can overwhelm antioxidant defenses and allow ROS to further react damaging the cell^6^. Plasma membranes are particularly susceptible to damage and as mitochondria are the greatest producers of ROS within the cell, high ppO_2_ is likely to cause mitochondrial membrane depolarization. One hypothesis is that this mechanism leads to endothelial dysfunction, which is exacerbated by the formation and growth of bubbles and is the primary cause of DCS symptoms^26, 39, 40^. Experiments on 2D cultures of endothelial cells have shown in real time that superoxide formation and consequent mitochondrial membrane depolarization occur during simulated dives^28^. In addition it has been shown that ROS scavengers such as N-Acetyl Cysteine (NAC) and dark chocolate reduce the incidence of DCS in rat and human divers^41, 42^. Whether this damage affects only endothelial cells and the extent that bubble formation exacerbates cellular dysfunction, remain unanswered research questions^28^.

Biomechanical/biochemical interactions have also been shown to occur between endothelial cells and bubbles. A series of works utilized 2D cultures of endothelial cells and an experimental system that brought microbubbles, generated at a pipette tip, into contact with the cells. The work was able to show that a mechanosensitive transmembrane ion channel (syndecan IV), was activated by bubble proximity due to the attraction of the hydrophobic side chains of the channel to the bubble air liquid interface. This activation resulted in a large calcium influx into the cell, ultimately resulting in cell death in 30% of endothelial cells which contacted a bubble^29–31^. Despite the clear connection of these results to DCS pathophysiology, the focus was once again on 2D cell cultures and decompression was not used to produce the bubbles. This research again raises the question as to whether other cell types, may be similarly sensitive to bubble proximity, and whether decompression will exacerbate the effect observed.

The aims of this work are to establish an experimental system capable of monitoring bubble dynamics in real time within a biomimetic tissue model. The biomimetic models should include a cellular population and be controllable and reproducible in its material parameters. With such a system we aim to quantitatively investigate: the effect cellular metabolism has on bubble dynamics, whether cell surfaces may act as bubble nucleation sites, and the consequences of a bubble producing decompression upon cell viability.

## Results

### Experimental system development

**Figure 1:**
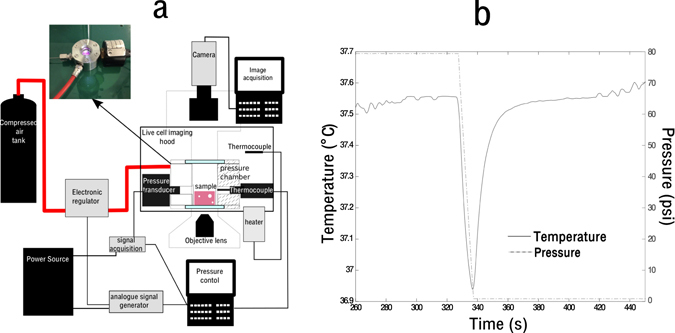
(**a**) Showing the experimental set up of the pressure chamber, with an image showing the chamber in working configuration, (**b**) showing the change in temperature with pressure variation in the chamber.

The experimental system designed to investigate extravascular bubble dynamics, consisted of a microscope compatible pressure chamber which could hold a single cylindrical sample of a type I collagen hydrogel. This system allowed the application of a pressure regimen to a collagen gel (acellular or cellular), and simultaneously visualize bubble formation and subsequent dynamics. Figure 1a shows the experimental set up. The temperature of the chamber was controlled via a PID heating system and an independent thermocouple to monitored air temperature variation within the chamber. Small temperature fluctuations of <1°C in the air of the chamber were seen during the most severe decompressions as shown in Fig. 1b.

These gels’ transparency in conjunction with the optical windows of the pressure chamber enables the imaging of bubble nucleation, growth and dissolution ‘within’ rather than on the surface of the gel. This combination of bubble imaging and biomimicry achieves the aim of investigating the dynamics of bubbles and their interactions with cellular structures.

**Figure 2:**
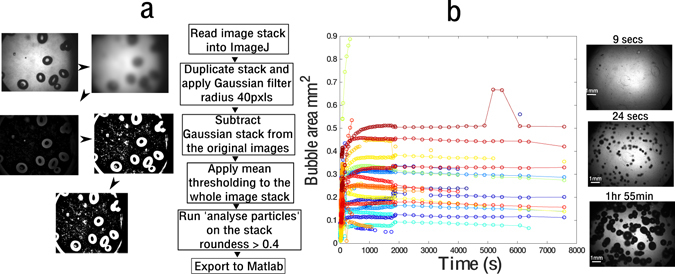
(**a**) Showing the segmentation and analysis process of bubbles that nucleate within the gels as a result of decompression from higher external pressure. (**b**) Showing representative images taken post decompression and a graph of the individual bubble radial trajectories that can be extracted from the images with the image anaylsis software.

Figure 2 shows the image analysis system with the segmentation routine (a) and the extraction of bubble radial trajectories (b). Statistical analysis of the radial time courses was performed by fitting an exponential growth expression to each individual radial time-courses via a robust non-linear regression (GraphPad). The plateau radius and the time taken to reach half the plateau radius were used to characterize the radial trajectories, and compare bubble growth dynamics across the experimental conditions (see Supplementary Figure 1 for details).

### Cellular metabolism and dissolved oxygen concentration

#### Increasing the tissue phantom cell density significantly decreases the dissolved oxygen concentration for phantoms with 500,000 cells/ml

**Figure 3:**
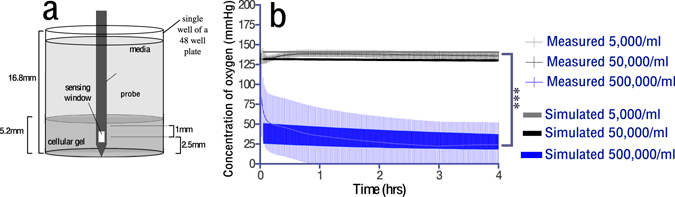
(**a**) The experimental setup, (**b**) the measured dissolved oxygen concentrations and the corresponding simulated dissolved oxygen concentrations for the area equivalent to the sensor window. Simulation error bars are due to repeated simulations (3 per cell density) with variation in the cell density within 10% of stated value, in addition to error caused by averaging over the sensor area. Other parameters values were D = 2.5 × 10^−9^ m^2^s^−1^ V_max_ = 3 × 10^−17^ mol cell^−1^ s^−1^, K_m_ = 5.6 mmHg, cell densities were 5,000 cells/ml, 50,000 cells/ml, and 500,000 cells/ml. Statistical significance calculated via a two way ANOVA with Tukey multiple comparison correction.

The hypothesis for this experiment was that increasing cellular density within the tissue phantoms would decrease the dissolved oxygen concentration. Dissolved oxygen concentration for tissue phantoms of increasing cell density were quantified using an invasive fluorescent probe embedded within the phantom (Fig. 3a).

The measured and simulated dissolved oxygen concentrations of tissue phantoms with three differing cell densities, are shown in Fig. 3b. A significant decrease in the measured dissolved oxygen concentration is seen for 500,000 cells/ml as compared to 5,000 and 50,000 cells/ml (p = 0.0003 and 0.0002 respectively). No significant difference was measured between 5,000 cells/ml and 50,000 cells/ml. Computational simulations of the experimental setup (Fig. 3a) were performed through the implementation of eq. (4) using values for *V*_*max*_ and *K*_*m*_ found in previous work^43^. The results of these simulations show good agreement to the measured values for all cellular densities. The higher measurement error in the experimental samples when compared to the modelled data is likely due to an inhomogeneous distribution of the cells within the gel and the relative position of the probe, as well as small movements in the probe within the gel.

### Nucleation and growth of bubbles

#### Bubble nucleation is significantly decreased in the highest cell density phantoms, but no statistical change in bubble plateau radii or half-lives were found with increasing cell density

Based on the literature, two competing hypotheses regarding the effect of increased cell density upon bubble nucleation can be proposed: (i) An increase in nucleation sites, provided by cell surfaces, will increase bubble nucleation^26^; or (ii) increased cell density will decrease the gas tension and hence reduce bubble nucleation^44^. Given previous work in this system utilizing various cell types^45^, it was hypothesised that oxygen metabolism rather than the presence of additional cell surfaces would dominate the resultant bubble nucleation.

**Figure 4:**
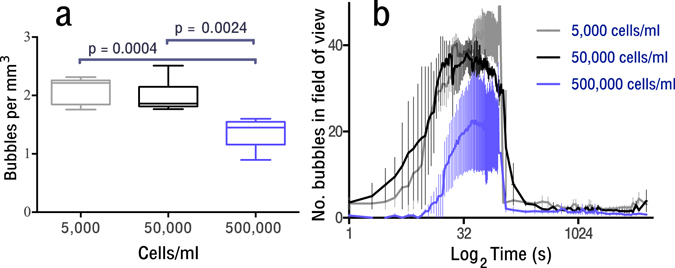
Nucleation of bubbles within cellular tissue phantoms containing 5,000, 50,000 or 500,000 HDF cells/ml. (**a**) The maximum number of bubbles which nucleated (median and min-max range shown), statistical significant was found between 500,000 vs. 5,000 cells/ml and (p = 0.0004) 500,000 vs. 50,000 cells/ml (p = 0.0023) as assessed by one way ANOVA (Tukey’s multiple comparison correction), (**b**) the variation in the number of bubbles in the field of view over time (N = 3 tissue phantoms per cell density).

Bubble nucleation was manually quantified for each phantom as described in the methods section. Figure 4 shows both the maximum (4a) and the change over time (4b) in the number of bubbles in the field of view. As shown in Fig. 4a, a significant decrease in the number of bubbles that nucleated was found between the 500,000 cells/ml group by comparison to the 5,000 cells/ml and 50,000 cells/ml groups (p = 0.0004 and p = 0.0024 respectively).

**Figure 5:**
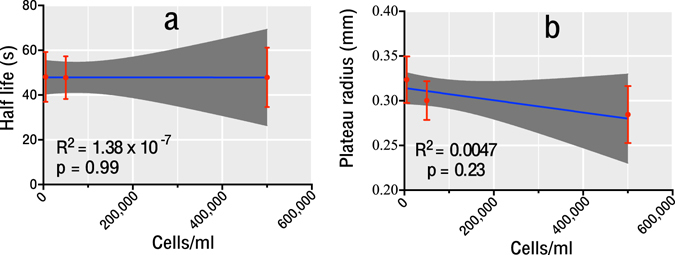
(**a**) Relationship between half life and cell density, (**b**) relationship between plateau radius and cell density, mean and 95% confidence intervals shown for points and for the linear regression fit. R^2^ and p-values of the linear regression given on each graph. No statistical dependence was found for either measured variable with varying cell density.

Bubble growth metrics for the same experiments are shown in Fig. 5. As can be seen no trend was found in either the mean bubble half lives or plateau radii with increasing cell density. This result contradicted our hypothesis that a reduction in the dissolved oxygen concentration would result in smaller bubbles (smaller plateau radius and shorter half life). In addition the R^2^ values in all cases are very small indicating that variation in the data cannot be explained by cellular density.

### Cell viability

#### Cell viability significantly decreases following a bubble-forming decompression

It was hypothesised that significant cell death would be measured following a bubble-forming decompression. In addition, the magnitude of cell death was hypothesised to be greater in our system than reported in the literature for 2D systems as both the biomechanical and increased *pp*O_2_ mechanism could act on the cells in our tissue phantoms.

**Figure 6:**
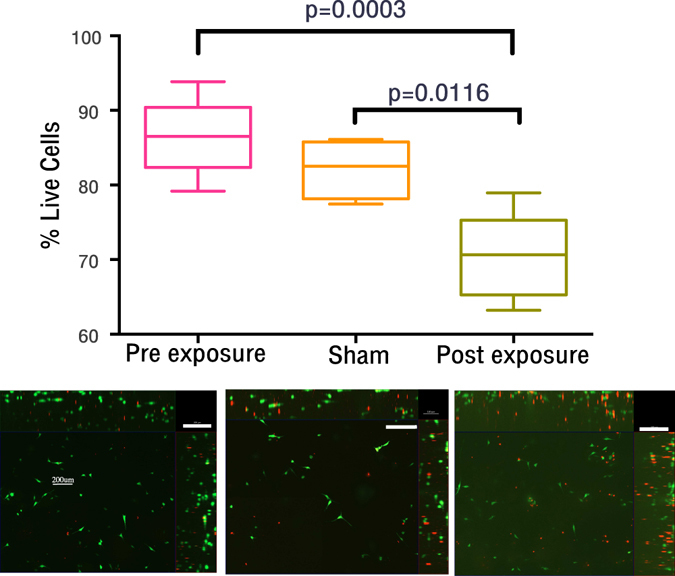
Comparison of the % of live cells in collagen hydrogels with 50,000 HDF cells/ml. Statistically significant differences are shown between the pre and post exposure (p = 0.0003) as well as the sham and post exposure (p = 0.0116). Significance assessed by ANOVA (Tukey’s multiple comparison correction), below the graph are representative images of the 3 conditions scale bars are 200 nm, red fluorescence indicates presence of ethidium homodimer in dead cells, green fluorescence indicates calcien-AM in live cells, N = 6. % cell death calculated by manual counting using formular % Live cells = calcien positive cells/(calcien-AM positive cells + ethidium homodimer positive cells).

To investigate the affect increased ppO_2_ and bubble formation had on cell viability fluorescent live dead staining measurements of the cells pre, post sham and post real pressure profile were taken. Tissue phantoms containing 50,000 cells/ml were used, as this cell density did not substantially decrease bubble nucleation and provided ample cells for accurate live dead analysis. Figure 6 shows the results of the fluorescent live dead analysis.

**Figure 7:**
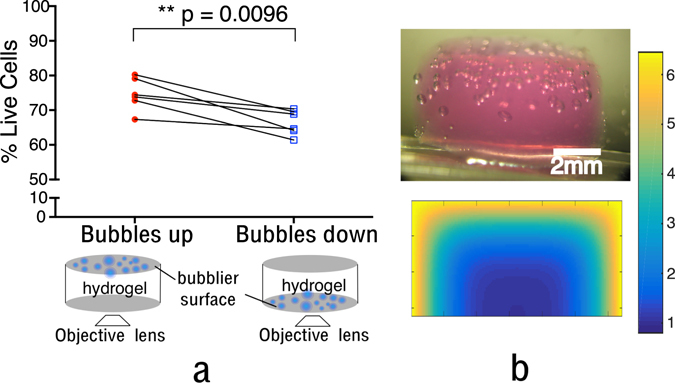
(**a**) The result of the post exposure live dead measurement spilt according to the orientation of the gel. Diagram of the orientation is shown below the plot. A statistical difference is found between the two orientations as assessed by a paired t-test (p = 0.0096 N = 6), (**b**) Upper image shows a cross-sectional view of a tissue phantom post dive, an inhomogeneous distribution of bubbles can be seen. Lower image shows a mid-point cross section of the simulated dissolved gas concentration within the phantom just prior to decompression i.e. (time 0 secs in Fig. 2b). The values have been non-dimensionalised to the atmospheric pressure. Simulation parameters are detailed in the methodology.

As can be seen there was a significant reduction in the proportion of live cells following the dive profile. The magnitude of the reduction in number of live cells was 11.56% and 15.82% from the sham and pre-dive respectively (p = 0.0116, p = 0.0003 respectively). Figure 7a shows the post exposure data separated according to the tissue phantom’s orientation. This was done as it was observed that the distribution of bubble formation appeared inhomogeneous (Fig. 7b (upper image)), where more bubbles formed towards the upper surface of the gel. As fluorescent imaging could not penetrate the entire thickness of the phantom, its orientation would influence whether cells imaged were more likely to be in contact with bubbles and the level of *ppO*_*2*_ cells had been exposed to. As the microscope was inverted, the “Bubbles up” case images lower surface of the tissue phantom, visa versa for the ‘Bubbles down’ case. Figure 7b (lower image) also shows the simulated dissolved gas concentration just prior to decompression. It can be seen that the distribution of bubbles appears qualitatively similar to the dissolved gas distribution, and that the upper surface of the phantom is exposed to a higher concentration of dissolved gas.

A significant difference between the two orientations was found (p = 0.0096), means and standard deviations were 74.63 ± 4.67% and 66.49 ± 3.601% for ‘Bubbles up’ and ‘Bubbles down’ respectively, corresponding to a maximum reduction in cell viability for the ‘Bubbles down’ case of 15.66% and 19.92% from the sham and pre-exposure respectively.

## Discussion

### Advantages and limitations of the experimental system

The experimental system developed in this work provides a much-needed tool for the investigation of extravascular bubble dynamics and the possible cellular interactions that may follow. The system can provide quantitative data on bubble growth and dissolution in real time, within a biomimetic engineered tissue phantom.

Other similar systems within the field have focused on one of three areas: Physical parameters, full animal or *ex vivo* models of tissue and 2D cell culture. The physical models include the gelatin model popularized by Yount *et al*.^44^ and utilised by Kunkle^46^, saline used by Van liew^47^, and Agarose of D’Arringo^48^. Despite their utility in providing data for underlying dive algorithm validation, the gelatin and other similar models are of questionable biological relevance and in the DCS literature have never been combined with live cell population. In addition, the pressure chambers previously reported for these models do not have the fine pressure control or time lapse imaging to investigate bubble dynamics in response to small variations in dive parameters. The other systems include hyperbaric chambers with optical windows into which an animal model or *ex vivo* tissue sample may be placed^49–51^. Such models suffer from the practical problem of being unable to image bubbles within the mostly opaque tissues. In addition such samples will naturally have a higher variability and for *ex vivo* tissues many of the surface bubble behaviours will be dominated by the cut surface topology^50^. Such models also represent an extreme of complexity as compared to dive algorithms and consequently data from such models is difficult to use in algorithm validation or development. Similarly it is difficult to use such models to establish mechanistic biological responses at a cellular level. The final type of experimental system utilizes 2D cell culture. Such setups are generally well suited to understanding mechanistic cell responses and provide highly controlled systems. However, the major drawback of such systems are their failure to capture the inherently 3-dimesional nature of bubble-cell interactions. Bubble dynamics cannot be captured in a 2D system and it is widely know that many cells cultured in 2D on plastic or glass, display altered cellular response to a range of stimuli^52^. Cells within a 3D collagen gel are in a more protective environment with cell-matrix adhesion in more complex geometric arrangements compared to cells attached to a 2D surface. Such gels have been used extensively within the tissue engineering field for many years^53^. Their efficacy in this field is largely due to excellent biocompatibility, the conservation of collagen across species as well as the control over cellular behaviours which can be engineered^54–57^. There is also a body of literature available on cellular metabolism, migration and proliferation^53, 58^. One drawback in the use of hydrogels is their relative mechanical weakness in comparison to tissues. Reported Young’s (E) and shear (μ) moduli for collagen hydrogel gels vary widely (E = 0.5-27kPa^59^, E = 1057 ± kPa^60^) and (μ = 0.003–0.08 kPa^61^, μ = 0.3–0.35 kPa^62^), but are in general lower than the majority of soft tissues E = 80 GPa for bone, to 1–2 GPa for a tendon, and 1–2kPa for the liver. μ = 1 Pa for the eye to 1–2 MPa for cartilage^63^. Strategies exist for increasing the mechanical stability of these gels^45^, however our present study was not focused on tissue elasticity and thus did not utilize these techniques.

Such a model we therefore believe provides an optimal level of complexity for producing quantitative data relevant to dive algorithm validation and development.

### Oxygen metabolism

To the best of the authors’ knowledge this is the first time that the relationship between cellular density, dissolved oxygen concentration and bubble nucleation and growth dynamics has been quantified. The decrease in bubble nucleation is of particular interest as it contradicts the proposal that cell surfaces act as nucleation sites *in vivo*, a hypothesis that is prevalent in the DCS field^26, 64^. The agreement of the modelled and measured dissolved oxygen (DO) concentration is of importance for two reasons: Firstly it shows validation of the Michaelis-Menten model, a simple kinetic model that could be relatively easily incorporated into a dive algorithm. Secondly, this validation allows further simulations to predict dissolved gas concentration in other cellular densities and to predict the gradients of dissolved gas within the current gels. Figure 7b (lower image) shows the spatially explicit dissolved gas concentration for a mid-point cross-sectional view of a tissue phantom with 50,000 cells/ml. Further results for differing cellular densities, metabolic rates and time points are shown in Supplementary Figure 2.

The cell densities used in this work are lower than those typically found *in vivo*, for example articulate cartilage is reported to have densities of ~2.5 × 10^6^ cells/ml and cortical bone 400,000–900,000 cells/ml^65^ both of which are relatively cell sparse tissues. However, our use of HDF’s in this work, is to create a variation on DO that is comparable to the *in vivo* case. *In vivo* DO is not a function solely of the cellular density, but also of the intrinsic cellular metabolic rate and the perfusion level of the tissue. Cartilage is populated by relatively quiescent chondrocytes and has measured DO of between 10.5 mmHg and 27.6 mmHg (in human septal cartilage), owing to its avascularity^66^. Other *in vivo* measures of various tissues show a wide range of values 32 mmHg for bone marrow^67^, 75 mmHg for rat skin and 57 mmHg for rat liver^68^. The most striking feature of the literature regarding DO measurment *in vivo* is the within tissue variation. In bone marrow for instance, reported values are as low as 9.9 mmHg. Given the relationship shown between DO and bubble nucleation in this work we suggest that variation in tissue DO should be more fully modelled in dive algorithms. As suggested by Van Liew^36^, the gradient of DO within tissues could impact the pattern of extravascular bubble formation. In other work such patterns have been remarked upon from invasive *in vivo* findings. Shupak *et al*. noted that bubbles in the rat mesenterium formed in a ‘distinct pattern’ mainly around the capillary network^69^. These authors did not provide their own explanation for the pattern and it is possible that the pattern reflected the dissolved gas concentration in the tissue.

The absence of a trend in the plateau radius or the half lives of the bubbles conflicts with the original hypothesis that smaller bubbles will form in more cell rich gels. Our explanation of this finding is that competition for dissolved gas amongst the bubbles affects the growth rate and counteracts any trend for smaller radii in more cell-rich gels. It has been shown in previous investigations in this system^45^, that where higher numbers of bubbles nucleate, the plateau radii are smaller and half lives shorter. This effect has also been recognized in the modelling literature, though not widely validated by quantitative bubble measurement^70^.

### Cell viability decreases as a result of bubble contact and increased *ppO*_*2*_

The results reported in Fig. 6 support the hypothesis that HDF cells would be susceptible to cell death and damage as a result of simulated dives. Two mechanisms by which cell death may be caused, bubble proximity and increased *ppO*_*2*_ have already been investigated by other researchers in 2D. For these mechanisms the reported magnitudes of cell death were 30% and ~20% respectively^28, 33^.

We have shown that significant cell death (p = 0.0003 and p = 0.0116) is seen in our 3-dimensional systems where both mechanisms (increased ppO_2_ and bubble contact) may act simultaneously. The magnitude of the cell death in our system (11–15%) compared to reported results in the other 2D systems, initially appears to show that the cell death in 3D is less than that in 2D. As shown in Fig. 7a, the orientation of the tissue phantom significantly changes the measured % of cell death, increasing it to 19.8–15.6% (from pre-dive and sham respectively) for the upper surface of the tissue phantom (p = 0.0096). The orientation results highlight the importance of performing experiments in a more realistic 3-dimensional system.

Within the 3D tissue phantom the distribution of bubbles appears to be non-homogeneous (Fig. 7b) with more bubbles towards the upper surface of the gel. Using the validated Michaelis-Menten kinetics to simulate the dissolved gas distribution in the phantom it can be seen that there is a gradient of dissolved gas through the phantom, which is qualitatively similar to the spatial distribution of bubbles (See Fig. 7b). Such gradients are also likely to exist in the extracellular space surrounding blood vessels *in vivo* ^36^. Both the DO gradient and bubble distribution mean that the proportion of cells exposed to the maximum ppO_2_ and those likely to experience bubble contact is far lower than in either of the 2D cell cultures. Estimating the number of cells in our system that contact bubbles is not possible with the current imaging setup however if one estimated that 50% of cells were in contact with a bubble, and used the literature reported 2D values of 30% predicted cell death in cases of contact; cell death in the 3D system would expected to be 15%^33^. The same is true for the *pp*O_2_, as seen in Fig. 7b. Only cells towards the edge of the tissue phantom are exposed increased *pp*O_2_, again if an estimate that 20% of cells are exposed levels of *pp*O_2_ comparable to the 2D experiment only a 4% increase in cell death would be expected. When such geometrical considerations are taken into account the % of cell death in the 3D system will be closer to, and possible greater than, those observed in the 2D systems. Improvements to the experimental system, such as a side window and separate imaging system could be used to provide accurate 3D bubble/cell positions. This would enable a more precise quantification of likelihood of cell death caused by bubble proximity and high ppO_2_ orientation.

In summary, this work has demonstrated the utility of an experimental system, which combines a small microscope compatible pressure chamber and collagen gel tissue phantom. It has been shown that increasing the cellular density of the gels and thereby decreasing the DO concentration, led to a decrease in the number of bubbles that nucleated. This result indicates that cell surfaces do not appear to provide suitable bubble nucleation sites. These nucleation results, although not directly applicable to *in vivo* tissue, are of use in defining parameter ranges for nuclei density, and for identifying probable nucleation sites *in vivo*. That no significant change in the plateau radii or half lives of the bubbles could be measured with varying cellular density (p = 0.23 and p = 0.99 respectively), highlights the importance of competition for dissolved gas as a mechanism to regulate bubble dynamics. Such interactions are considered only in small number of DCS bubble models^38, 70–72^. In addition, widely used dive algorithms do not vary the magnitude of the oxygen windows in different tissue compartments^38, 71, 73^, however, as shown here, this may have a significant impact on the bubble density and hence bubble dynamics of different tissues. This is an effect that could be easily incorporated into current dive algorithms by use of Michaelis-Menten kinetics or by simply varying the fixed oxygen window size parameter for different tissue compartments. The significant increase in cell death that was measured following a bubble-forming decompression (p = 0.003 and p = 0.0116), demonstrates the direct effect that bubble formation and *pp*O_2_ have on cell viability. Our data in 3D shows a decrease in decompression induced cell death compared to previously published data using 2D models. The emerging acceptance of 3D models as more biomimetic systems, supports the claim that our data provides a better representation of the *in vivo* case. The native collagen matrix will elicit more biomimetic cellular responses; and as discussed, the geometry of the system better reflects the *in vivo* environment. Although the geometry of the system may be considered trivial; in the case of DCS, this geometry has ramifications on the dissolved gas distribution, bubble distributions and dynamics; and therefore, larger scale cellular responses. It is not clear how such responses impact the pathophysiology of DCS when considering a human diver.

The potential for increasing the sophistication of the measurements made and the complexity of such biomimetic models in our experimental system, will enable cell-type specific, mechanism of dysfunction to be made going forward. Ultimately it is plausible that some of the wide-ranging symptoms of DCS could be understood from such investigations.

## Methods

### Experimental system development

The pressure chamber was designed in AutoCAD and manufactured in stainless steel with two sapphire glass windows (Edmund optics) in the upper and lower surfaces. The chamber was designed and tested to sustain a maximum operating pressure of 145 psi, with the chamber pressure independently monitored by two pressures transduces (QVP1, and DSX Proportion-Air). A maximum decompression rate was 13 psi/s and pressure profiles were defined on a 0.5 s time intervals. The chamber pressure is electronically programmed using the pressure regulator (QVP1 Proportion air) via a Labview interface and time-lapse images of the sample within the chamber taken via a microscope mounted camera (Hamamatsu C4742-95-12G04) in conjunction with HCL live imaging software.

Collagen hydrogels are transparent gels created by the fibrillogenesis of type I collagen extracted from rat-tail. Various cell types may be incorporated within the gels prior to fibrillogenesis and the water content of the gels can be varied, leading to variation in a gel’s material parameters^52^.

### Tissue Phantom construction

Human dermal Fibroblast HDF cells (Tissues were collected in 2001, anonymized at the point of collection and handled according to the relevant regulations and guidelines). Cells were used at passages 5–12 and project protocols were approved by the UCL/RNOH ethics committee. Cells were cultured in Dulbeccos modified Eagles medium (DMEM), 2 mmol/l glutamine high glucose, (Sigma, UK), with 10% foetal calf serum (FCS; First Link, UK) and penicillin streptomycin (500 unit ml^−1^ and 500 μml^−1^) (ICN Biochemicals, UK). Collagen hydrogel gels (0.5 ml) were made up of 0.4 ml collagen (0.1:10) polymeric collagen, to monomeric collagen (rat tail collagen type I (First Link, UK)). Polymeric collagen was extracted as per the protocol in Wong *et al*.^74^. Briefly, approx. 1 cm^3^ samples of calf tendon (obtained from an abattoir) were frozen and weighed. Each sample was placed in a pestle and mortar with a small amount of liquid nitrogen and crushed to break up the tendon, until a powder was produced. The tendon powder was then added to 0.5 M EDTA (Gibco) (100 ml/g of tendon) and stirred overnight at 4 °C. The solution was then centrifuged at 200 rmp for 2 mins and the supernatant removed. The 0.5 M EDTA was replaced and the mixture stirred for 4 hrs at 4 °C. The solution was washed with distilled water twice. Acetic acid 0.5 M (100 ml/g of tendon) was then added to expand the collagen. This was stirred overnight at 4 °C. To purify the polymeric collagen, 0.1 ml of Modified Eagle’s Medium (MEM) (Gibco, UK) per 1 ml of solution was added to the acetic acid collagen mix, and 1 M NaOH used to neutralise the mixture whilst simultaneously stirring with a cold glass or metal rod. The unidirectional shearing created by the stirring, caused polymeric collagen to accumulate on the rod. The polymeric collagen was then transferred to fresh acetic acid (0.5 M) and stirred overnight at 4 °C. The purification process was repeated twice.

A blended gel was made by the addition of polymeric collagen (6.85 mg/ml) to monomeric collagen (2 mg/ml), at a ratio of 0.1:10 polymeric to monomeric collagen. This resulted in a final mixture with a concentration of 0.0685 mg/ml polymeric collagen. Added to this collagen mixture was, 0.05 ml 10X concentration Modified Eagle’s Medium (Gibco, UK). This mixture was neutralised by the drop wise addition of 5 M NaOH and then stored on ice for 1hr to remove excess bubbles. After 1 hr 0.05 ml of cells suspension with the required cell density was added. Cellular densities were calculated by adding in a ratio of 1:1 2:1 or 4:1, Trypan blue to cell suspension; 10 μl of this mixture were introduced to the haemocytometer and cells counted manually with a dissecting microscope. Two counts were made for each cell density and the number averaged. Gels were then pipetted into a 48 well-plate (0.5 ml per well) and allowed to set at 37 °C for 15 mins. After this a further 0.5 ml of DMEM was added to the top of the gels and these were left for 8–12 hrs at 37 °C.

### Oxygen monitoring

A 48 well plate was placed a jig within a hypoxia incubator. Oxygen probes were attached to the jig ensure placement of probes was consistent. Media was removed from the gel surface and fresh media replaced (0.7 ml). An oxygen probe (NX-NP/O/E Oxford Optronix) was inserted into the center of the gel vertically until the tip of the gel contacted the well plate base see Fig. 3a. Oxygen measurements were taken every 80 secs for 8 hrs or until there was an obvious problem with a probe reading at which time the experiment was terminated.

For bubble growth experiments, cellular gels were transferred from a well to the pressure chamber using a needle to detach the gel from the well edge and a spatula to lift the gel under aseptic conditions. 0.5 ml of DMEM media was added to the pressure chamber. A pressure profile was applied with initial pressure increase at 1 psi/s until a max pressure of 80 psi, 30 mins was then spent at the maximum pressure prior to a decompression at 8 psi/s. Time-lapse imaging commenced from the start of the decompression at 1 sec intervals for 100 images then 30 secs for 50 images, followed by 12 images at 5 mins intervals and a final image at 2 hrs. Cell viability measurements were made of cellular gels subjected to: the previously described pressure profile, a sham dive where the gel was transferred to the chamber for the same total time with no pressure applied, or where the gel was not disturbed from the 48 well plate. Following the profile end, gels were immediately transferred to a universal tube containing 17 μl ethidium homodimer (2 mM; Invitrogen) and 20 μl calcein-AM (4 mM; Fluka Analytical) diluted in 5 ml PBS, and incubated for 35 mins. Gels were then imaged on Carl Zeiss Apotome inverted fluorescence microscope. 6 images of each gel were taken, 3 on one side at 0, 120 and 240^◦^ the gel was then turned over and the procedure repeated. In each case 200 μm total depth was imaged in slices of 6 μm thickness. Live dead cell percentages were manually counted for all samples.

### Numerical modelling

Numerical modelling was performed using a finite difference approach. The dissolved gas concentrations for both O_2_ and N_2_ were described via discretized versions of eq. (4) (*V*_*max*_ = *0* for the case of N_2_). Simulations were performed in a combination of C++ and Matlab. A three-dimensional Cartesian lattice of nodes was used to represent the discretized dissolved gas concentration of a tissue phantom. The dissolved gas concentration at every time point *(t)* was described for each gas *(g)*, at each node in Cartesian co-ordinates $C{(t)}_{(i,j,k)}^{g}$. Equation (4) was non-dimensionalised as previously described^45^ and solved using the Alternative Direction Explicit finite difference scheme^45, 75, 76^ with a time-step *Δt* = *2 secs* and a spatial step *Δh* *=* *0.17* *mm*, set according to stability requirements. It was assumed that the bulk diffusion coefficient was homogeneous as was the distribution of cells. Two different boundary conditions were imposed on the different sides of the phantom according to the experimental case being considered. For the real-time oxygen monitoring simulations: base–no flux, vertical sides–no flux, top–Dirichlet. For the decompression simulations: base–no flux, vertical sides–Dirichlet, top–Dirichlet. The Dirichlet values were specified from Henry’s law (1) and the known ambient pressure. In the case of the real-time oxygen monitoring simulations, phantoms were assumed to initially be in equilibrium with the ambient pressure $C{(0)}_{(i,j,k)}^{{O}_{2}}={k}_{h}^{{O}_{2}}pp{(0)}^{{O}_{2}}$. The same condition (with appropriate parameter changes) was assumed for nitrogen. Total dissolved gas concentration is calculated by the addition of dissolved N_2_ and O_2_ at each node_._ For the decompression case the initial conditions for each node in the 3D lattice was taken from the output of the real-time oxygen monitoring simulations.

### Data Availability

The datasets generated during and/or analysed during the current study are available from the corresponding author on reasonable request.

## Electronic supplementary material

Supplementary information
