# Supplementary material for: Quantification of cell-bubble interactions in a 3D engineered tissue phantom
Source: Sci Rep. 2017 Jul 24;7:6331. doi: 10.1038/s41598-017-06678-y (PMC5524813; doi:10.1038/s41598-017-06678-y)
Supplement: Supplementary file 1 — Supplementary information [file 41598_2017_6678_MOESM1_ESM.pdf]

Quantification of cell-bubble interactions in a 3D engineered tissue phantom

C. Walsh[1,2,3], N. Ovenden[3], E. Stride[4], U. Cheema[2],

[1]Centre for Mathematics and Physics in the Life Sciences and Experimental Biology (CoMPLEX), UCLPhysics BuildingGower StreetLondon, WC1E 6BT.

[2] UCL Institute of Orthopaedics and Musculoskeletal Science, London, UK.

[3] Department of Mathematics, University College London, London, UK

[4]Institute of Biomedical Engineering, Old Road Campus Research Building, University of Oxford, Oxford UK.

Supplementary Information

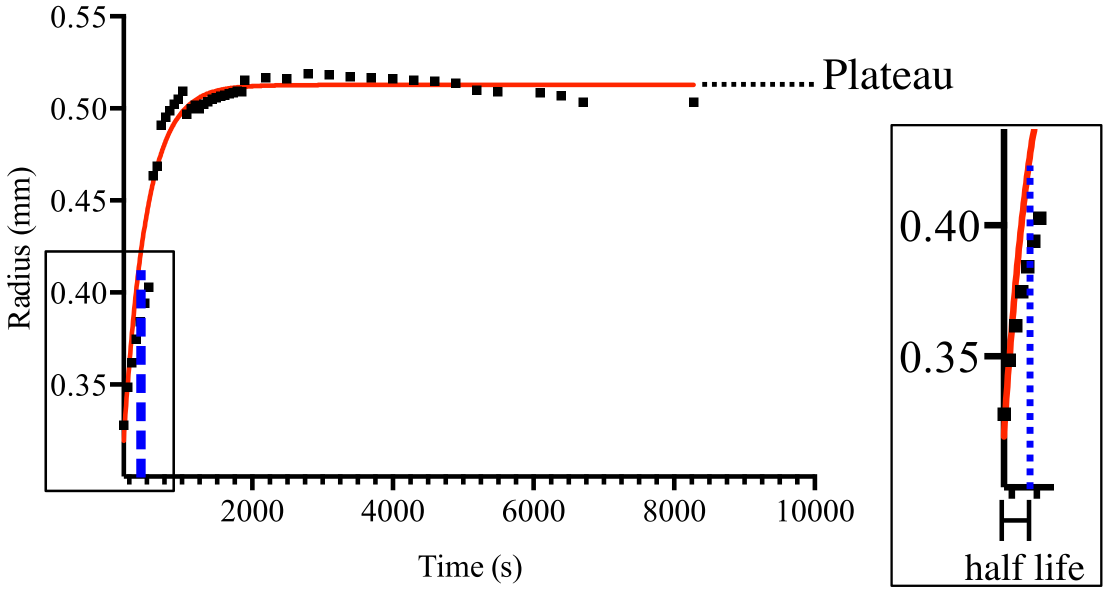

Figure SI1 shows an example of the non-linear regression analysis for a single bubble's radial trajectory. Black dots show measured bubble radius, red line shows the non-linear fit. The half-life is shown in the inset, and plateau is marked on the main plot.

To compare the dive parameters each individual bubble time course was analysed via non-linear regression in Graphpad version 6. The regression model chosen was a single-phase exponential decay equation described by:

$$R = R_0 - Plateau^{-t\ln 2/\tau} + Plateau\ ,$$

where R is the radius R_0 is the initial radius, τ is the half life, and Plateau is the asymptotic value of the radius. The fit was constrained to ensure the plateau value was greater than the initial radius. A robust regression method was employed as the residuals were not normally distributed even after a log transform. From the regression analysis the values of the plateau and half life were used to characterise each bubble trajectory and the mean values to compare between experimental conditions.

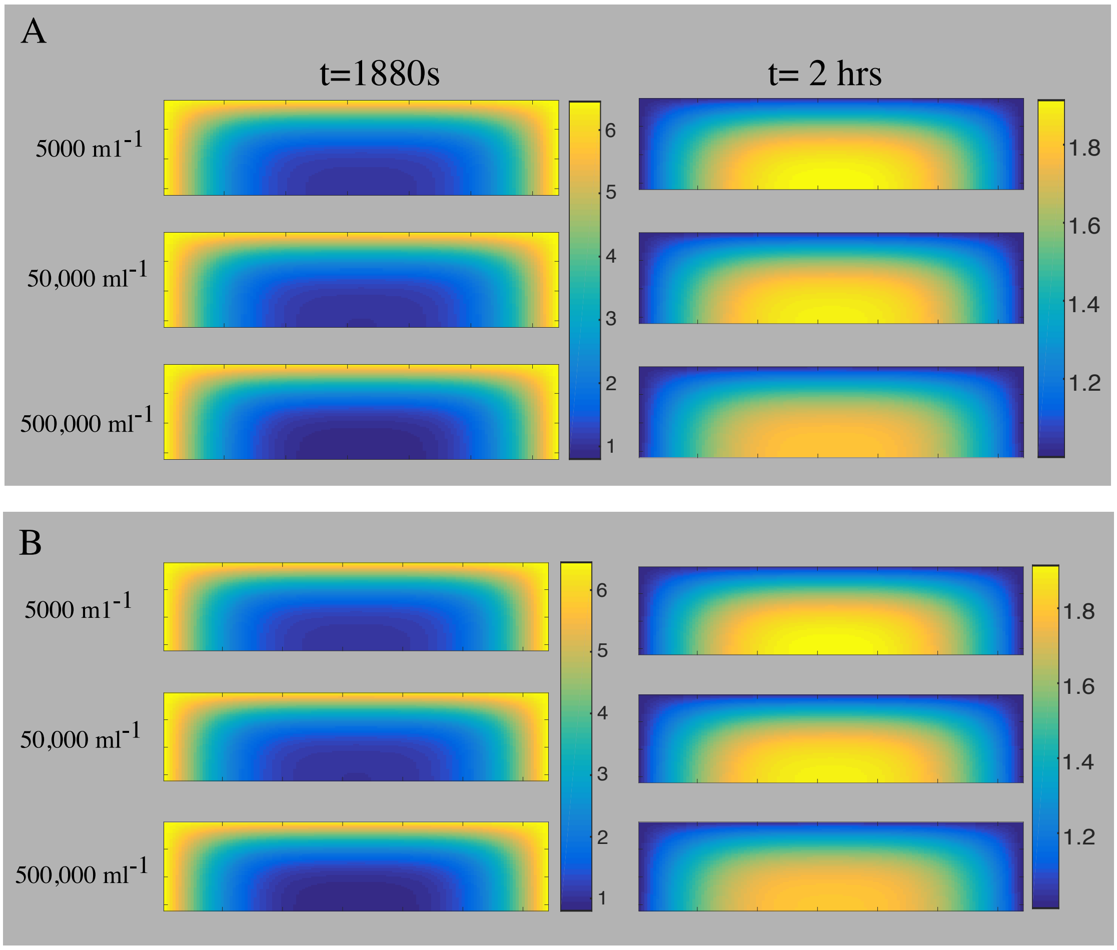

Figure SI 2 Showing the simulated dissolved oxygen concentration for the mid-point cross section of the tissue phantoms just prior to decompression (left), and at 2hrs after the start of the profile (right). (A) shows the case where cellular metabolism continues at the same rate throughout the pressure profile, (B) shows the case where all cellular metabolism stops when pressure profile begins. Concentrations for all plots use the scale shown in the scale bar on the right-hand side, where all concentrations have been non-dimensionalised to atmospheric pressure.
